# Enhancing Maternal Outcomes by Standardizing Epidural Analgesia Practice: A Quality Improvement Project in a Secondary Hospital in Taif City

**DOI:** 10.7759/cureus.109021

**Published:** 2026-05-17

**Authors:** Alaa M Abdelhafeez, Fahad K Alomari, Bassam A Alzaidi, Rehab Al-Talhi, Abdullah Alalyani, Mustafa Suliman, Mohamed Mansy, Latifah Algethami, Cristina Abner, Ahmed Alsawat

**Affiliations:** 1 Department of Anesthesia, Prince Sultan Military Hospital, Taif, SAU; 2 Department of Anesthesia, Omdurman Islamic University, Khartoum, SDN; 3 Department of Family Medicine, Prince Sultan Military Hospital, Taif, SAU; 4 Department of Continuous Quality and Patient Safety, Prince Sultan Military Hospital, Taif, SAU; 5 Department of Medical Education, Hamad Medical Corporation, Doha, QAT; 6 Department of Obstetrics and Gynaecology, Prince Sultan Military Hospital, Taif, SAU; 7 Department of Obstetrics and Gynecology, Prince Sultan Military Hospital, Taif, SAU

**Keywords:** cesarean section rate, epidural labour analgesia, maternal outcomes, multidisciplinary collaboration, obstetric anesthesia, patient satisfaction score, secondary care hospital, taif city

## Abstract

​Introduction

A critical service gap was identified at a secondary military hospital in Taif City, characterized by periods of zero utilization of epidural analgesia (EA) and an elevated baseline cesarean section (CS) rate. This quality improvement (QI) project was initiated to recover EA services, reduce maternal operative morbidity, and improve clinical outcomes through a standardized multidisciplinary practice change.

​Materials and methods

Using a quasi-experimental (retrospective-prospective) design, clinical metrics from the baseline period (January-September 2024) were compared with the post-intervention period (January-September 2025). A multidisciplinary team (anesthesia, obstetrics, nursing, and administration) implemented a three-cycle Plan-Do-Study-Act (PDSA) framework. Interventions included root cause analysis, resource optimization, and data-driven monthly performance huddles. Patient satisfaction was assessed via a prospective cross-sectional comparative study.

​Results

Following the intervention, EA utilization significantly increased from 4.9% (n = 30/610) in 2024 to 13.5% (n = 85/628) in 2025 (χ² (1, N = 1,238) = 26.24, p < .001; Cramer's V = 0.15). The CS rate decreased from 40.2% (n = 410/1,020) to 32.5% (n = 302/930) (χ² (1, N = 1,950) = 12.19, p < .001; Cramer's V = 0.08), while spontaneous vaginal deliveries (SVD) increased from 55.9% (n = 570/1,020) to 63.8% (n = 593/930) (χ² (1, N = 1,950) = 13.06, p < .001; Cramer's V = 0.08). Instrumental delivery rates remained stable at 3.9% (n = 40/1,020) and 3.8% (n = 35/930), respectively (χ² (1, N = 1,950) = 0.15, p = .698). In 2025, patients receiving EA reported significantly higher satisfaction (93.6%; n = 80/85) compared to the non-EA group (76.5%; n = 415/543) (t (626) = 35.15, p < .001). Mean staff satisfaction during implementation was 74.6% (n = 47/63).

Conclusions

This standardized, consultant-led model successfully optimized labor analgesia and delivery outcomes, providing a resilient and reproducible framework for enhancing maternal safety and institutional resource stewardship at a national scale.

## Introduction

Childbirth is an intensely painful experience, and providing effective pain relief is crucial for improving the maternal experience. A growing body of research suggests that fear of labor pain is a key factor in rising Cesarean delivery rates. Increasing the utilization of epidural analgesia (EA)--the gold standard for managing labor pain--can help reduce unnecessary cesarean sections (CS). EA not only provides superior analgesia but also facilitates instrumental deliveries and surgical conversions, which is especially beneficial for high-risk patients such as those with obesity. Experts, such as Akadri and Odelola, advocate for offering labor analgesia to all women who request it to optimize clinical outcomes [1-3].

​A recent study at our institution (2023) revealed an exceptionally low EA utilization rate of 5.3%, contrasting sharply with other regions in Saudi Arabia. This trend was primarily attributed to a significant lack of awareness, with only 6.7% of antenatal patients familiar with EA, a figure substantially lower than the 16.2% to 85.6% awareness range reported elsewhere in the Kingdom. Furthermore, patient attitudes remained mixed; while some women expressed interest, many remained resistant due to a combination of cultural beliefs and a lack of evidence-based information [4].

​Educating expectant mothers about EA is a vital strategy for fostering informed decision-making and improving overall attitudes toward labor pain management. By proactively addressing concerns and increasing awareness of effective relief options, healthcare providers can significantly reduce the prevalence of elective CS motivated by a fear of pain, ultimately empowering women to navigate labor with greater confidence [5]. ​Ultimately, offering EA provides significant benefits, including a superior patient experience and the prevention of unnecessary cesarean deliveries, which carry their own set of major health risks for mothers [6].

By September 2025, this initiative aims to achieve measurable excellence by reaching three targets: increasing EA utilization to 20%, reducing the total CS rate to 30%, and achieving a patient satisfaction score exceeding 90%.

## Materials and methods

Study design, setting, and population

This quasi-experimental, pre- and post-intervention study was conducted in the seven-bed Labor and Delivery (L&D) unit at Prince Sultan Military Hospital. The eligible population comprised all parturients admitted for intended vaginal delivery during the January to September periods of 2024 (n = 610) and 2025 (n = 628). Inclusion was restricted to patients with cervical dilatation between 3 cm and 7 cm. Patients with pre-planned cesarean deliveries, those presenting outside the specified dilatation range, or those with medical or obstetric contraindications to EA were excluded. Data from the 2025 intervention period were benchmarked against institutional baseline performance from the corresponding months in 2024. Additionally, a cross-sectional comparative design was utilized to assess satisfaction levels between patients receiving EA and those opting for alternative analgesia during the 2025 study period.

​Interventions and quality improvement framework

​The project utilized a three-cycle Plan-Do-Study-Act (PDSA) framework to standardize and scale the EA service.

PDSA cycle 1 (Re-launch): This established foundational infrastructure by implementing the lowest effective dose analgesia (LEDA) utilizing a standardized low-dose infusion of 0.0625%-0.1% bupivacaine with 1-2 mcg/mL of fentanyl at rates of 4-12 mL/hour, launching an antenatal patient education program via brochures and posters, and stabilizing the supply chain through a minimum-stock notification system.

PDSA cycle 2 (Breakthrough): The second cycle achieved a clinical breakthrough by focusing on multidisciplinary expansion and resource optimization. We doubled service capacity through a strategic nursing cross-training initiative to resolve labor ward staffing shortages. To drive sustainability, obstetricians spearheaded patient education, with completion validated by objective comprehension assessments. Technical proficiency was strictly enforced via verified logbooks, requiring anesthesiologists to demonstrate 10 procedures with ≥85% patient satisfaction and zero major complications. This phase was anchored by continuous consultant oversight and targeted re-education to ensure institutional high-performance benchmarks.

**Figure 1:**
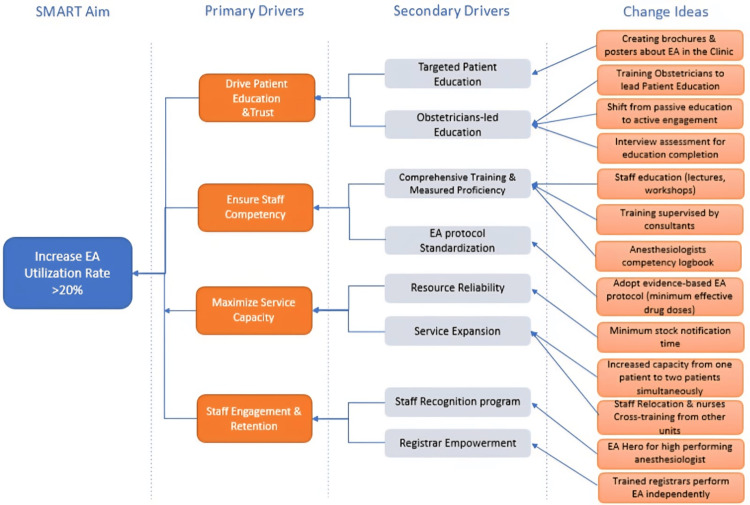
Driver diagram summarizing the interventions EA: epidural analgesia

PDSA cycle 3 (Sustainability): This targeted long-term service continuity amidst critical consultant staffing constraints. This was achieved through strategic task-shifting, authorizing proficient registrars for independent practice to ensure 24/7 coverage, and the implementation of a peer-recognition "EA Hero" program to incentivize high safety and patient satisfaction metrics (Figure 1).

During the initial phase, staffing constraints limited EA to one patient at a time, requiring a triage strategy that prioritized primigravida patients and those requesting a cesarean delivery due to labor pain anxiety. The second PDSA cycle successfully expanded capacity to two simultaneous patients; however, further growth was restricted to ensure clinical safety amid ongoing staffing shortages.

​Data collection and outcome measures

​Performance data were reviewed during mandatory monthly huddles and validated by the institutional Continuous Quality Improvement and Patient Safety (CQI & PS) department. ​Data were triangulated from electronic databases and digital surveys, utilizing patient-facing QR codes and SMS links alongside staff questionnaires via WhatsApp-based platforms. Primary outcome measures included the EA utilization rate, total CS rate, and patient satisfaction scores. Process and balancing measures focused on education program completion rates, staff satisfaction scores, and the rate of major clinical complications associated with EA.

​Statistical analysis

​Data management and analysis were performed using Microsoft Excel, Version 2024 (Microsoft Corp., Redmond, WA) and jamovi, Version 2.6.44 (The jamovi project, 2024). Figures and graphical representations were further refined using Canva (Canva Pty Ltd, Sydney, Australia). Categorical variables, such as CS and EA rates, were analyzed using the chi-square test to determine statistical differences between pre- and post-intervention periods. For comparative analysis of numerical data, paired t-tests were utilized. A p-value of P ≤ 0.05 was considered statistically significant for all analyses.

## Results

Participant flow and delivery characteristics

A total of 1,950 deliveries were analyzed over the two-year study period, comprising 1,020 deliveries in 2024 and 930 deliveries in 2025. Delivery modes were categorized into three groups: spontaneous vaginal delivery (SVD), CS, and instrumental delivery (ID).

​The data indicate a year-over-year shift in clinical outcomes, characterized by a decrease in the proportion of surgical interventions and a corresponding increase in the rate of vaginal deliveries. These primary delivery metrics, which serve as the baseline for the subsequent analysis of EA utilization and clinical pathway efficacy, are detailed in Table 1.

**Table 1:** Distribution of maternal delivery outcomes (2024–2025) ​N: Total number of deliveries; n: Number of patients within a specific sub-category *Instrumental delivery includes vacuum-assisted, forceps, and other assisted vaginal delivery methods.

Delivery Metric	2024 (N = 1020)	2025 (N = 930)
Cesarean Section	40.2% (n = 410/1020)	32.5% (n = 302/930 )
Spontaneous Vaginal Delivery	55.9% (n = 570/1020)	63.8% (n = 593/930)
Instrumental Delivery *	3.9% (n = 40/1020)	3.8% (n = 35/930)
Total Deliveries	100% (1020)	100% (930)

Outcomes measures 

The primary outcome measures assessed in this study were the EA utilization rate, the CS rate, patient satisfaction scores, and the overall financial impact.

Epidural analgesia utilization rate

**Figure 2:**
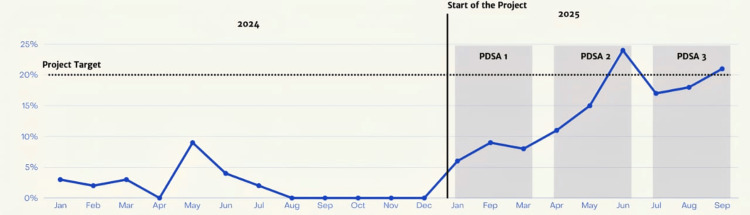
Trends in epidural analgesia (EA) utilization rates across PDSA cycles during 2024 and 2025 PDSA cycle: plan-do-study-act cycle

​The utilization of EA demonstrated a statistically significant increase following the intervention. In 2024, out of 610 total vaginal deliveries (SVD and ID cases), EA was administered in 30 cases (4.9 %). In 2025, following the service intervention, EA utilization rose to 85 cases out of 628 total vaginal deliveries, representing a rate of 13.5%. A chi-square test of independence confirmed this increase was highly significant, χ² (1, N = 1,243) = 26.15, p < .001, with an effect size of Cramer's V = 0.15. Analysis of monthly aggregate data (n = 9 months) further supported this, showing a significant increase in the mean monthly utilization rate from 3.33% (SD = 3.64) to 9.44% (SD = 7.42), t (8) = 7.72, p < .001 (Figure 2).

​CS rate

**Figure 3:**
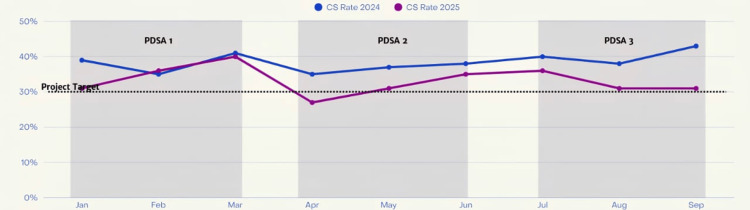
Comparison of total cesarean section (CS) rates between 2024 and 2025 CS: cesarean section; PDSA cycle: plan-do-study-act cycle

A statistically significant reduction in the CS rate was observed during the study period. The total proportion of CS procedures decreased from 40.2% (n = 410/1,021) in 2024 to 32.5% (n = 302/930) in 2025. This population-level reduction was statistically significant, χ² (1, N = 1,951) = 12.19, p < .001, with an effect size of Cramer's V = 0.08. On a month-to-month basis over a 9-month period (n = 9), the mean monthly CS rate decreased from 38.4% (SD = 8.11) in 2024 to 33.1% (SD = 5.90) in 2025, t (8) = 14.71, p < .001 (Figure 3).

In parallel, the total proportion of SVD increased from 55.7% (n = 569/1,021) in 2024 to 63.8% (n = 593/930) in 2025. This increase was statistically significant, χ² (1, N = 1,951) = 13.06, p < .001, with an effect size of Cramer's V = 0.08. On a month-to-month basis, the mean monthly SVD rate improved from 55.7% (SD = 4.12) to 63.8% (SD = 5.43), t (8) = 27.02, p < .001, demonstrating a successful shift toward vaginal deliveries following the intervention.

Instrumental deliveries remained statistically unchanged between 2024 and 2025. The total proportion was 4.1% (n = 42/1,021) in 2024 and 3.8% (n = 35/930) in 2025 χ² (1, N = 1,951) = 0.15, p = .698). The mean monthly rate was 4.4% (SD = 2.19) and 3.9% (SD = 1.90), respectively, t (8) = -2.03, p = 0.077.

​Patient satisfaction

**Figure 4:**
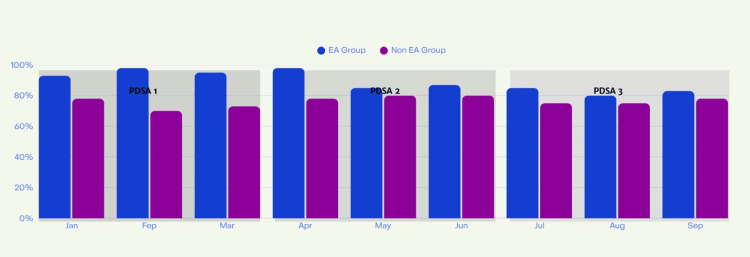
Comparison of patient satisfaction scores between EA and non-EA groups in 2025 ​EA: epidural analgesia; PDSA: plan-do-study-act cycle

​In 2025, patient satisfaction scores varied significantly according to intrapartum pain management, with the EA group (n = 85) demonstrating a significantly higher mean satisfaction score of 93.6% (SD = 3.9 %), whereas the non-EA vaginal delivery group (n = 543) reported a mean satisfaction score of 76.5% (SD = 3.1 %), t (626) = 35.15, p < .001 (Figure 4).

Economic impact

​As shown in Table 2, the implementation of the practice change resulted in a reduction of 108 CS between January and September 2025 compared to the same period in 2024 (302 vs. 410). Based on an institutional cost range of $4,250 to $5,989 per procedure, this decrease generated an estimated total cost saving of $459,000 to $646,812 USD [7]. These results underscore the significant fiscal benefits of enhanced resource stewardship within the obstetric unit.

**Table 2:** Economic impact of reduced cesarean section rates (January to September 2024 vs. 2025) No: number; USD: United States Dollars

Metric	Value/ Range
Study Period	January - September ( 2024 & 2025)
Average Cost per Single Cesarean Section (USD)	$4,250 - $5,989
No. of Cesarean Sections (2024)	410
No. of Cesarean Sections (2025)	302
No. of Reduced Cesarean Sections	108
Total Estimated Cost Savings (USD)	$459,000 - $646,812

​Balancing measures

​To assess the broader impact of the practice change on the clinical environment and ensure that the primary improvements did not negatively affect other areas of care, both staff satisfaction and the rate of major complications were monitored.

​Staff satisfaction

**Figure 5:**
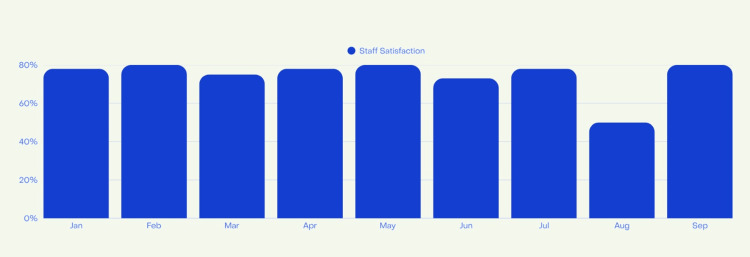
Monthly staff satisfaction rates from January to September 2025

Staff satisfaction for the 2025 implementation period yielded a mean score of 74.9% (n = 63, SD = 0.085), with monthly satisfaction scores ranging between 53.0% and 80.0% (Figure 5).

Rate of major complications

​The major complication rate remained at 0% for the majority of the study period. A single adverse event occurred in February involving an unintended intravenous infusion of epidural medication. The patient experienced transient dizziness consistent with mild local anesthetic systemic toxicity (LAST) but remained clinically stable due to the use of a low-concentration local anesthetic protocol. Following this event, immediate corrective actions and nursing re-education were implemented to mitigate future risk.

Process measures

They track the completion rates for both professional staff training and patient education programs.

​*Staff Training Program Completion*

**Figure 6:**
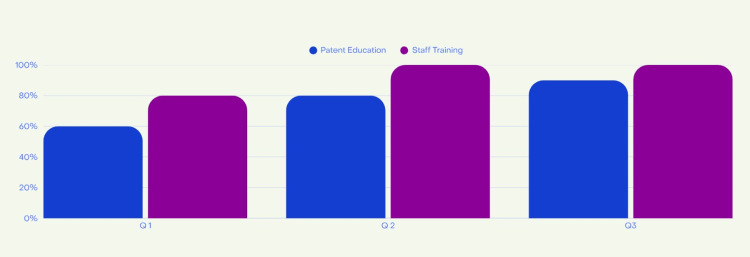
Comparison of staff training and patient education completion rates from Q1 to Q3 2025 Q: quarter

Staff training program completion rate demonstrated a consistent increase from 80% (n=50/63) at the conclusion of the first quarter (Q1) to a sustained 100% (n=63/63) throughout the second and third quarters (Q2 and Q3) (Figure 6).

​*Patient Education Program Completion*

​The completion rate for the patient education program followed a similar upward trajectory, rising from 60% (n=390/650) in Q1 to 80% (n=520/650) in Q2, and reaching 90% (n=585/650) by the end of Q3 (Figure 6).

## Discussion

The findings of this project underscore a transformative shift in labor analgesia and its broader obstetric implications. While EA remains the definitive benchmark for intrapartum pain management [3], our results demonstrate that a consultant-led, standardized practice model serves as more than a clinical service; it acts as a strategic framework to optimize the birthing experience while safely modulating surgical intervention rates.

​Impact on delivery mode and cesarean rates

​A persistent concern in obstetrics has been the potential association between EA and an increased rate of CS, a debate fueled by earlier literature suggesting that EA may substantially increase the risk of surgical delivery [8]. However, our data provides evidence to the contrary. By adopting a lowest effective dose (LED) drug policy and standardized practice changes, a statistically significant reduction was observed in the total proportion of CS, which declined from 40.2% (n = 410/1,021) in 2024 to 32.5% (n = 302/930) in 2025 following the implementation of the standardized practice.

​This outcome is particularly noteworthy, as it surpassed the national benchmark of 35% [9]. In Saudi Arabia, fear of pain-absent primary medical or obstetric indications accounts for approximately 15% of CS cases [2]. Therefore, the drop in the mean CS rate suggests that providing effective, standardized pain relief can directly mitigate the demand for elective surgical intervention. This aligns with regional data from Jeddah, which reported that EA is not associated with increased emergency CS or adverse maternal outcomes [10].

Obstetric literature historically links EA to an increased risk of operative vaginal birth, as the association between its use and a protracted second stage has reinforced the clinical assumption that superior analgesia inevitably necessitates mechanical intervention [11,12]. Conversely, our findings demonstrate that standardized clinical workflows and rigorous oversight can facilitate a significant increase in analgesia uptake while maintaining stable ID rates. This suggests that a dose-optimized, policy-driven approach effectively mitigates the labor delays traditionally observed in conventional models, confirming that high levels of maternal comfort can be achieved without an escalation in assisted deliveries when managed within a consistent framework.

​Regional variations and neonatal safety

​The impact of EA appears more pronounced in the Taif region, suggesting that addressing "fear of pain" through education is a critical driver of vaginal delivery rates. Furthermore, the safety profile of EA is reinforced when compared to systemic alternatives. Research indicates that neonates whose mothers receive intravenous or intramuscular opioids require more naloxone and exhibit lower Apgar scores [13]. By prioritizing standardized EA, we achieved superior maternal comfort and optimized the delivery trajectory without adverse impact on neonatal safety, an approach associated with a 35% reduction in the risk of severe maternal morbidity (SMM) as supported by current literature [14].

​Sustainability, value, and future directions

Although the service did not meet international accessibility benchmarks for neuraxial analgesia, defined as 90% of requests fulfilled within 30 minutes, the initiative successfully reached the 90% patient satisfaction benchmark [15] and demonstrated significant clinical and financial value, generating annual cost savings between $459,000 and $646,812 while ensuring sustainability through institutional standard operating procedures (SOPs) and automated electronic health record (EHR) audits. ​To facilitate broader adoption and replication, we developed a comprehensive adoption package. This includes a registrar competency logbook, an obstetrician’s educational manual, and a detailed blueprint of all interventions used throughout the project. These tools convert localized success into a nation-ready template for other secondary healthcare facilities. While this project focused on a single center, the next phase involves multi-site validation to establish a national standard of care. Future goals include maintaining at least a 30% EA utilization rate and expanding this proficiency framework to other high-risk obstetric procedures, ensuring long-term improvements in maternal safety across the Kingdom.

Study limitations and system resilience

Execution within a secondary care setting introduced specific operational constraints, including fluctuations in staffing levels and periodic supply chain disruptions. These challenges were addressed through an early notification system and a structured peer-recognition initiative to ensure service continuity. While such variables may affect the immediate generalizability of the model to different institutional tiers, the framework’s success despite these disruptions underscores its systemic resilience and utility in real-world, resource-constrained environments.

​Additionally, cultural barriers to neuraxial analgesia among specific demographics may have introduced a degree of selection bias. While a targeted, obstetrician-led educational model was employed to mitigate this, the findings may not fully reflect the breadth of all patient perspectives across diverse socio-cultural backgrounds. Furthermore, technical barriers necessitated a transition from singular QR-code access to diversified digital feedback channels. Although this broadened the response pool, the reliance on self-reported metrics remains a limitation common to quality improvement research, potentially subject to response bias.

## Conclusions

Integrating a consultant-led, standardized labor epidural analgesia practice into hospital policy catalyzes a sustainable paradigm shift in maternity care. This model effectively eliminates clinical variation and prevents major complications, significantly increasing epidural analgesia utilization and reducing cesarean section rates without escalating instrumental deliveries. Even amidst resource shortages, the framework demonstrates remarkable system resilience, yielding superior clinical and economic outcomes alongside high patient satisfaction. These findings offer a scalable, high-value template for national implementation across the Kingdom to optimize maternal safety and resource stewardship.
